# Somatostatin Receptor Theranostics for Refractory Meningiomas

**DOI:** 10.3390/curroncol29080438

**Published:** 2022-08-04

**Authors:** Betty Salgues, Thomas Graillon, Tatiana Horowitz, Olivier Chinot, Laetitia Padovani, David Taïeb, Eric Guedj

**Affiliations:** 1 APHP, Pitié-Salpêtrière Hospital, Nuclear Medicine Department, Sorbonne University, 75013 Paris, France; 2 INSERM, MMG, APHM, Timone Hospital, Neurosurgery Department, Aix-Marseille University, 13005 Marseille, France; 3 APHM, Timone Hospital, Nuclear Medicine Department, Aix-Marseille University, 13005 Marseille, France; 4 APHM, Timone Hospital, Neuro-Oncology Department, 13005 Marseille, France; 5 APHM, Timone Hospital, Radiotherapy Department, 13005 Marseille, France; 6 CERIMED, APHM, Timone Hospital, Nuclear Medicine Department, Aix-Marseille University, 13005 Marseille, France; 7 CNRS, Centrale Marseille, Institut Fresnel, CERIMED, APHM, Timone Hospital, Nuclear Medicine Department, Aix-Marseille University, 13005 Marseille, France

**Keywords:** meningioma, peptide receptor radionuclide therapy, somatostatin receptor, treatment-refractory meningioma

## Abstract

Somatostatin receptor (SSTR)-targeted peptide receptor radionuclide therapy (PRRT) represents a promising approach for treatment-refractory meningiomas progressing after surgery and radiotherapy. The aim of this study was to provide outcomes of patients harboring refractory meningiomas treated by 177Lu-DOTATATE and an overall analysis of progression-free survival at 6 months (PFS-6) of the same relevant studies in the literature. Eight patients with recurrent and progressive WHO grade II meningiomas were treated after multimodal pretreatment with 177Lu-DOTATATE between 2019 and 2022. Primary and secondarily endpoints were progression-free survival at 6-months (PFS-6) and toxicity, respectively. PFS-6 analysis of our case series was compared with other similar relevant studies that included 86 patients treated with either 177Lu-DOTATATE or 90Y-DOTATOC. Our retrospective study showed a PFS-6 of 85.7% for WHO grade II progressive refractory meningiomas. Treatment was clinically and biologically well tolerated. The overall analysis of the previous relevant studies showed a PFS-6 of 89.7% for WHO grade I meningiomas (*n* = 29); 57.1% for WHO grade II (*n* = 21); and 0 % for WHO grade III (*n* = 12). For all grades (*n* = 86), including unknown grades, PFS-6 was 58.1%. SSTR-targeted PRRT allowed us to achieve prolonged PFS-6 in patients with WHO grade I and II progressive refractory meningiomas, except the most aggressive WHO grade II tumors. Large scale randomized trials are warranted for the better integration of PRRT in the treatment of refractory meningioma into clinical practice guidelines.

## 1. Introduction

Meningiomas represent 30% of the primary brain tumors. They are associated with heterogeneous clinical outcomes [1]. Approximately 80% of these tumors are benign (grade I according to WHO grade) and the remaining cases are related to WHO grade II and III (collectively classified as high-grade meningiomas (HGMs) [2]. Treatment options mainly encompass neurosurgical resection and therapeutic radiation. After complete resection, the 5-year recurrence rate is estimated to be 5%, 40%, and 80% for grade I, grade II, and grade III, respectively [1,3]. The incidence of recurrence has been associated to the extent of resection (Simpson grade), localization, high grade, and more recently Ki-67 expression level or molecular signatures (mutations in SMO, AKT1, and the TERT promoter) [2,4,5,6]. If HGMs exhibit an aggressive behavior characterized by high recurrence rates and resistance to standard treatments, the management of benign meningiomas can also be challenging in complex anatomical localizations with an increased risk of morbidity and incomplete treatment. When no further surgical or radiotherapeutic options are available, the role of systemic therapy remains unclear and experimental in any grade of meningioma [3,7]. New treatment approaches are therefore required for these patients. Peptide receptor radionuclide therapy (PRRT) has gained an increasing role over the past 20 years in the field of neuroendocrine tumors (NETs) and represent an attractive approach for the treatment of meningomas. 

Indeed, Graillon et al. showed that all categories of meningiomas overexpressed somatostatin receptor subtype 2 (SSTR2) mRNA, at levels similar to those of human soma-totrophic pituitary adenomas or GEP-NETs and independent of their grade [8]. 

Positron emission tomography (PET) imaging with somatostatin analogues (SSTa) radiolabeled by Gallium-68 has gained an increasing role in tumors with somatostatin receptors expression. Previous studies showed a higher sensitivity of 68Ga-DOTA-SSTa PET/CT than MRI in the detection of tumor tissue for de novo meningiomas, recurrence, and especially osseous involvement [1,9]. New MRI studies are improving diagnostic performance, including better distinction between meningiomas and schwannomas [10].

Rachinger et al. have shown a strong correlation between tumor SUVmax (standardized uptake value) and semi-quantitative SSTR2 (immunostaining) in meningioma [11]. 

Again, no relationship was found between SUVmax and WHO grade. In addition to the diagnostic aspect, the “theranostic” aspect of 68Ga-DOTA-SSTa PET/CT is crucial to select patients eligible for PRRT. By exchanging the radionuclide, the same tracer can be used either for diagnostics or for therapy (PRRT) using 90-Yttrium or 177Lutetium-DOTATATE. Several studies have evaluated the role of PRRT in meningioma patients [12,13,14,15,16,17,18]. A main advantage of PRRT over external beam radiotherapy relies on its molecular selectivity for meningioma cells and its ability to treat multicentric lesions within the same session.

The present work aims to provide an overview of PRRT with 177Lu-DOTATATE for refractory progressive meningiomas with the following elements: a retrospective study, an overall analysis of progression-free survival at 6 months (PFS-6) of the same relevant studies in the literature stratified according to WHO grade, and prospects for the development of this therapeutic approach.

## 2. Materials and Methods

### 2.1. Patient Population

All patients with progressive refractory meningioma treated with 177Lu-DOTATATE between August 2019 and October 2021 at La Timone University Hospital, France were included in this retrospective study. Inclusion criteria for PRRT were progressive meningiomas with no further surgical or EBRT treatment options and SSTR-positive lesions with equal or greater tumor uptake than liver on pre-therapy DOTATOC PET scans. Patients were excluded if they met one of the following criteria: lesion with mass effect on the brain stem, urinary incontinence, preexisting grade 3 or 4 hematologic toxicity, or ECOG performance status ≥ 3. Eligibility was screened during a neuro-oncological tumor board meeting at La Timone University Hospital and more recently during the national French meningioma tumor board meeting. Informed consent was obtained from all patients for compassionate use of PRRT in meningioma. Approval for the retrospective analysis was obtained from the institutional review board of neurosurgery (approval code: IRB00011687 date of approval 13 May 2022).

### 2.2. Imaging

68Ga-DOTATOC was performed before initiation of the first therapy cycle. PET/CT imaging were performed on a GE Healthcare Discovery PET/CT 710 (General Electric Healthcare, Waukesha, WI, USA) and/or Siemens Biograph TruePoint 16 (Siemens Healthcare, Erlangen, Germany) and/or 5-Ring GE Healthcare Discovery MI PET/CT. A whole-body imaging was acquired at approximately one-hour post-injection (2–3 MBq/kg). A low dose CT scan for anatomical correlation and attenuation correction was performed. Contrast-enhanced MRI was performed every three months. Post-therapy 177Lu-DOTATATE scintigraphy was obtained 24 hours post-injection on a Siemens Simbia system (Siemens Heathcare, Erlangen, Germany).

### 2.3. Therapy

Radionuclide therapy was administered with the somatostatin analog DOTATATE labeled with 177Lutetium (Lutathera^®^, Novartis, Switzerland). Treatment was administered by a slow intravenous infusion during 30–45 min, in cycles of minimal 3200 MBq and maximal 7400 MBq per cycle for a maximum of 4 cycles in 8–9 week intervals. We also administered co-therapeutic agents commonly including steroids, anti-emetics, and infusion of amino acid solution for renal protection in line with previous studies evaluating SSTR-based radionuclide therapy. 

### 2.4. MRI Response Assessment

Response assessment was based on the RANO working group [19]. Accordingly, standard bidimensional measurements were performed on MRI. Progressive disease was defined as follows: ≥25% increase in bidimensional product. 

### 2.5. Clinical and Biological Examinations

Each therapy cycle was preceded by clinical examination in the department of neuro-oncology and/or nuclear medicine. Clinical tolerance was evaluated by assessing the evolution of motor or sensory disorders, the size of subcutaneous tumor masses or exophthalmos when present in certain patients, the frequency of epileptic seizures, and the dosage of corticosteroids. Biological tolerance was assessed by performing a blood test every 2 weeks during PRRT. At the end of the PRRT, the neuro-oncologist reviewed the patient every 3 months with an MRI and a blood test (liver, kidney, and hematological function).

### 2.6. Previous Studies

We considered studies that investigated of at least five patients treated with somatostatin analogues (SSTa) radiolabeled by 177-lutetium or 90-Yttrium in refractory or progressive meningioma. Refractory meningioma was defined as meningioma without control after surgery, radiotherapy and/or radiosurgery, and/or systemic therapy.

Several data were not available in the selected studies. The authors of two papers were contacted. One was unable to provide the requested data and the other did not answer. We were aware of potential bias due to missing data.

### 2.7. Outcomes

We extracted data on each published paper. We reported progression-free survival at 6 months (PFS-6), WHO grade. In all studies, PFS was estimated at the onset of first PRRT. Overall Survival (OS) is only of interest in randomized studies. As there is a lack of comparative historical data, OS was not reported.

### 2.8. Data Synthesis

We pooled all available necessary data, stratified by the WHO grade, and generated for PFS-6 an overall mean weighted by the number of patients in each study.

## 3. Results

### 3.1. Patients Characteristics 

**Figure 1:**
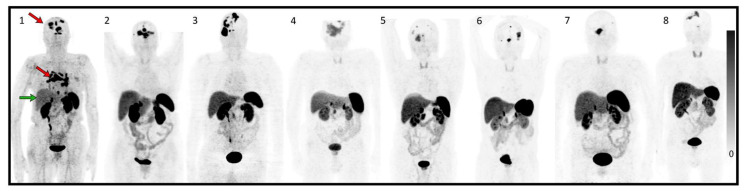
Maximum intensity projection (MIP) of 68Ga-DOTATOC before treatment of each patient. (**1–8**) correspond to patients treated in accordance with the rest of the article.The red arrows in patient **1** show intense uptake in all intra-cranial multifocal meningiomas and mediastinal lymph node metastases, greater than the uptake in the liver (green arrow). The remaining patients (patients **2–8**) have multifocal intracranial meninigiomas also showing higher or equal uptake of Ga-DOTATOC in tumor tissue than in healthy liver tissue.

Patient and pretreatment characteristics are summarized in Table 1. Eight patients harbored WHO grade II meningiomas according to the latest 2021 WHO classification [2]. Seven patients underwent four cycles of PRRT with 177Lu-DOTATATE (7.4 GBq/cycle) each 8–9 weeks and patient 1 underwent one and a half cycles of PRRT between August 2019 and October 2021. Median age was 72.5 years, and five out of eight were male. All patients showed high SSTR expression with Krenning score ≥ 2 graded by 68Ga-DOTATOC PET/CT (Figure 1). Patient 1 died 3 months after the first cycle of PRRT of tumor progression with major intracerebral edema.

**Figure 2:**
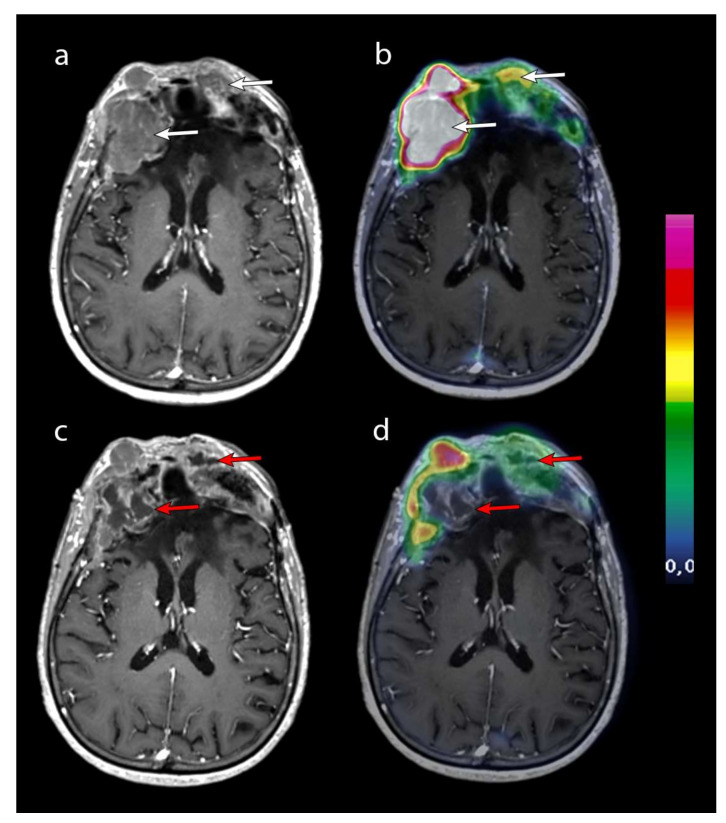
Contrast-enhanced T1-weighted axial MRI after the second cycle of PRRT (**a**) and after the fourth cycle of PRRT (**c**) was merged with 68GA-DOTATOC PET (respectively, panels (**b**) and (**d**)). Two frontal lesions (white arrows) with high 68GA-DOTATOC uptake necrotized after 4 cycles (red arrows). The question remains as to the origin of this necrosis, which may be a direct effect of PRRT or a natural necrotic tumor progression. Patient 2 was the only patient to progress with this necrotic appearance. Unequivocal progression of the other lesions was assessed according to RANO criteria on the MRI performed 4 months later.

An MRI was performed every 3 months. Data were incomplete for patient 7. Baseline MRI was performed between 8 days and 80 days before the start of PRRT. Our latest patients (patients 4 to 8) were well followed up with a baseline MRI performed between 8 days and 29 days before the start of PRRT (Figure 2).

According to bidimensional RANO criteria, six out of seven patients were stable 6 months after the first cycle of PRRT. MRI was not performed during follow-up for patient 7 (the 68Ga-DOTATOC lesion volumetry was stable, but the patient was excluded from the PFS-6 analysis). Follow-up of patient 6 was not long enough to evaluate him at 12 months. Five out of six patients were stable at 12 months. PFS-6 was 85.7% (*n* = 6/7) and PFS-12 was 66.7% patients (*n* = 4/6) (Table 2).

### 3.2. Toxicity

In our series, the treatment was well tolerated. One patient experienced early progression and died (patient 1). 

Biologically, the most common adverse effect was transient grade 1 hematotoxicity (anemia and/or thrombopenia, and/or lymphocytopenia, and/or leukopenia) according to the Common Terminology Criteria for Adverse Events (CTCAE v6.0). Patients 3, 6, and 7 experienced transient grade 3 lymphocytopenia. 

Patient 2 (85 years old woman) experienced grade 2 anemia, requiring blood transfusions and erythropoietin injection during and after the third cycle of PRRT, due to poor tolerance. She also experienced deterioration of her renal insufficiency the year following the PRRT (grade 2–3). Patient 3 retained grade 1 anemia 35 months after the first cycle of PRRT.

**Figure 3:**
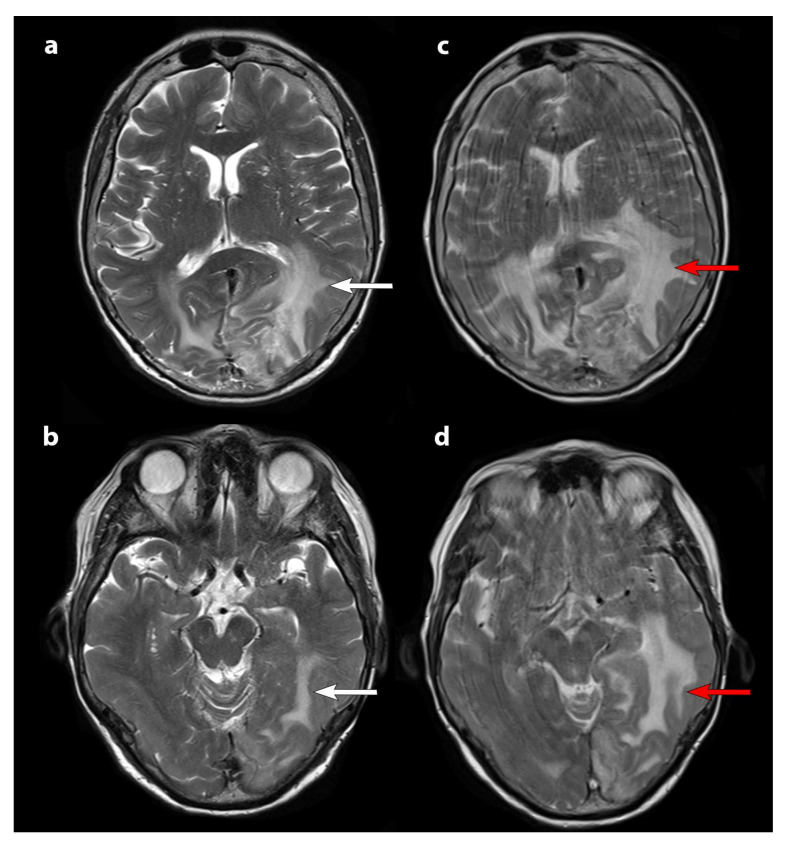
T2-weighted axial slice MRI. MRI before therapy (panels (**a**) and (**b**)) and after the second cycle of PRRT (panels (**c**) and (**d**)). White arrows show brain edema on baseline MRI which increases after the second cycle (red arrows). An increase in kinetic movements at the time of the second MRI (panels (**c**) and (**d**)) were due to the patient’s clinical deterioration in parallel with the radiological progression.

Clinically, the most frequent adverse event was asthenia. Two patients had more frequent transient seizures. With the exception of patient 1, increased edema on the MRI was not described in the other patients. This patient already harbored a significant brain oedema on baseline MRI; increased doses of cortisone were not sufficiently effective (Figure 3).

### 3.3. Study Selection

A total of eight relevant studies/trials were identified and selected in addition to our case series. Seven studies were included, evaluating radiolabeled somatostatin analogues alone and one study assessing combination of SSTR-targeted PRRT and fractionated EBRT in refractory progressive meningiomas [12,13,14,15,16,18,20]. The study from Kreissl et al. has been reported for information in Table 3 and not included in the analysis (patients were treated by EBRT and one cycle of 177Lu-DOTA-TATE).

Three studies were phase II clinical trials [14,18,20]. Studies from Parghane et al., Seystahl et al. Müther et al., were retrospective, and those from Bartolomei et al. and Van Essen et al. were prospective studies [12,13,15,16].

PFS data were not available from Marincek et al. and were not complete from Bartolomei et al. because of pooled data between WHO grade II and III meningiomas (*n* = 14). We excluded in the analysis all the cohorts from Marincek et al. (*n* = 34) and WHO grade II and III meningiomas from the Bartolomei et al. cohort in the analyses stratified by grade (*n* = 14). 

We evaluated a total of 86 patients among them, 29 patients had WHO grade I meningiomas (33.7%), 30 patients had WHO grade II (34.9%), 17 patients had WHO grade III (19.8%), and there were 10 patients with an unknown WHO grade (11.6%). 

### 3.4. Treatment Response

Response assessment was not homogeneous due to different radiological evaluation protocols: Two studies used RECIST 1.1 criteria (Response Evaluation Criteria in Solid Tumors version 1.1), one study used SWOG (Southwest Oncology Group) criteria, one study used Macdonald criteria, two studies used RANO criteria, and for 3 studies, assessment protocols were unknown. 

### 3.5. Month Progression-Free Survival according to WHO-grade

PFS-6 according to WHO grade was analyzed for 72 patients. The 6-month PFS was 89.7% for WHO grade I meningiomas (*n* = 29); 57.1% for WHO grade II (*n* = 21) and 0 % for WHO grade III (*n* = 12). For all grades (*n* = 86), PFS-6 was 58.1%. PFS-6 for unknown grades was 100%. Based on the available data and following RANO criteria, the best radiological response obtained was stable disease.

## 4. Discussion

Despite recent advances in targeted therapy or immunotherapy, recurrent high-grade meningioma non-accessible to surgery or radiation therapy remains an unmet medical need in neuro-oncology with a poor patient outcome [7].

Our present case series showed a PFS-6 of 85.7% for WHO grade II refractory progressive meningiomas using RANO 2D criteria for response assessment. The overall analysis of the previous relevant studies, including ours, showed a PFS-6 of 89.7%; 57.1% and 0%, respectively, for WHO grade I, II and III meningiomas. 

Kaley et al. in 2014 published a RANO review about outcome benchmarks for future medical therapy trials in meningiomas refractory to surgery and radiotherapy, and proposed a PFS-6 interest rate of >50% for WHO grade I and >35% for WHO grade II/III [22]. Recently, Mirian et al. published an individual patient data meta-analysis including all published meningioma patients treated with SSTR-targeted PRRT with three different radiopharmaceuticals [23]. Our results are slightly different from theirs but in agreement, PFS-6 was 94%, 48%, and 0% for WHO grades I, II, and III, respectively. 

The analysis of three new cohorts was added in the analyses of our study compared to the meta-analysis of Mirian et al.: ours, Müther et al., and Parghane et al. cohorts [15,17]. The cohort of Minutoli et al. was not included in the analyses because the radiopharmaceutical used was 111-indium, an auger particle emitter, in contrast to 90-Yttrium and 177-Lutetium, which emit beta particles [24]. The inaccessibility of separate data for WHO grade II and III meningiomas in the Bartolomei et al. cohort and separate data for all grades in the Marincek et al. cohort led to weaker results. These peculiarities of the analysis may explain the differences identified between Mirian et al. and our analyses of PFS-6 for each WHO grade.

Analysis of our cohort shows a 6-month PFS rate for grade II meningiomas that is higher than the rate reported in previous studies (85.7%) assessed with the 2D RANO criteria on MRI. These rates may be explained by the selection of patients who received this treatment. High SSTR expression (Krenning score ≥ 2) is not sufficient to select patients for PRRT in meningiomas. Hyperprogressive patients were not selected in our cohort because of the lack of potential substantial benefits of PRRT for this subgroup. Moreover, Graillon et al. reported that preinclusion growth rate impacts the treatment response of combining mTOR inhibitor everolimus and somatostatin agonist octreotide (response, stabilization, growth slowdown, and persistent growth) [25]. The same result regarding the impacts of pre-inclusion growth rate is expected with the PRRT. Once EBRT would no longer be possible, the identification of high-risk tumors and a sign of tumor activity could provide a basis for proposing PRRT earlier in the patient’s disease history. Waiting for a clear and rapid progression before proposing PRRT may in some cases lead to a risk of poorer patient selection (rapid progression rate before PRRT).

We do not routinely evaluate dual tracer molecular imaging (68Ga-DOTA-SSTa and 18F- fluoro-2-deoxy-D-glucose (FDG)), which could also play a key role in patients’ selection for PRRT in certain pathologies. 

There is still uncertainty as to whether large tumor volume is a predictor of poor response to 177Lu-DOTA-SSTa given the physical characteristics of 177-Lutetium. If the radionuclide distribution is uniform, optimal cure diameters extend from a minimum range of 1.2 mm to a maximum range of 3 mm for 177-Lutetium which is well below the size of the meningiomas we currently treat [26]. It is also questionable whether certain lesions would be more likely to respond to this type of treatment, such as lesions near the cavernous sinus that benefit from extensive drainage. 

Another uncertainty concerns the ability of 177Lu-DOTA-SSTa treatment to induce an antitumor immune response that reduces tumor escape and may explain the excellent therapeutic responses.

It can be argued that the efficacy of PRRT is more questionable in most aggressive and rapidly progressive meningiomas. The proposal of PRRT in this context will have to involve a strategy of early detection of this aggressiveness, optimization of PRRT, and/or therapeutic synergy.

Biologically, the most common adverse effect was transient grade 1 hematotoxicity (anemia and/or thrombopenia, and/or lymphocytopenia, and/or leukopenia) according to the Common CTCAE v6.0. Lymphocytopenia is one of the most common adverse events among hematotoxicities. For example, in the Gerster-Gilliéron et al. cohort, 8/15 had transient lymphocytopenia. In the Seystahl et al. cohor, lymphocytopenia affected 70% of patients, including severe (grade 3 and 4) lymphocytopenia in 30% of patients. 

Clinically, the most common adverse events were asthenia, worsening of the patients’ respective neurological deficits, and increased frequency of seizures. In the present cohort, peritumoral edema increase was clearly identified for one patient. Pseudo progression in patients treated with PRRT is debated in the community and is considered a rare phenomenon. Our first hypothesis was a multifactorial origin: the consequence of tumor progression and possibly the effect of PRRT. There were no other similar cases in our cohort with a significant increase in edema, however the introduction or increase in corticosteroid therapy improved the clinical situation of patients. The link with tumor volume is not clear. 

177Lu-DOTA-TATE intra-venous administration has been approved in SSTRs-positive well-differentiated gastroenteropancreatic neuroendocrine tumors (GEP-NETs), at a recommended fixed dose of 7.4 GBq every 8 weeks, for a total of four cycles. There are still outstanding issues due to the aggressiveness of refractory meningioma compared to NETs including whether 7.4 GBq every 8 weeks is the correct dosage to achieve the best possible response. 

The utility of internal dosimetry is not immediately apparent in metastatic NETs (mNETs) treated with radionuclide therapy, but there is a specific need in refractory meningiomas. If we want to move from a fixed dosage to a personalized dosage, internal dosimetry could provide answers regarding the determination absorbed radiation dose–effects relationships and the optimization of this therapeutic approach to guide personalized treatment. Unfortunately, internal dosimetry is rarely performed in clinical routine, with more difficulty in analyzing the data compared to external radiotherapy, and its usefulness is debated in the medical community, leading to a poor understanding of the theoretical empirical radiobiology in PRRT.

Another approach to optimize the efficacy of treatments is to modulate the route of administration. The intratumoral/intra-resected tumoral cavity injection by port-a-cath system (Rickham, or Ommaya reservoir) or radioembolization could be a solution as an adjuvant therapy modality increasing biological effect dose (BED) and reducing adverse effects. 

Rechallenge with one or more 177Lu-DOTATATE therapy cycles after four initial PRRT cycles can be proposed to these patients in therapeutic failure if PRRT is well tolerated and brings a clinical benefit or a stabilization of the disease over several months.

A study evaluating the combination of EBRT and one cycle of 177Lu-DOTA-SSTa was added in this paper [20]. The publication of Kreissl et al. was recently complemented by another publication 8 years after the first one to evaluate the long-term efficacy and toxicity of this combination. Combined EBRT and PRRT was well tolerated without severe or chronic toxicity. Disease stabilization has been shown in 7/10 patients with a median PFS of 107.7 months vs. 26.2 months for the three patients with meningioma progression (range, 13.8–75.9 months) [21]. EBRT and PRRT have different organs at risk (OARs), a combination of the two could improve the absorbed dose and provide better local disease control than PRRT alone while preserving the benefit-risk balance.

In addition, pre-clinical studies have shown that radiation exposure increases SSTR2 expression, which could be a promising concept to boost the antitumor effects of PRRT in aggressive tumors [27,28]. This opens the way for future prospective studies.

One of the other promising approaches is the use of radiosensitizing agent in combination with PRRT. In a phase I-II study investigating the safety and efficacy of radiopeptide 177Lu-octreotate combining with capecitabine and temozolomide in advanced low-grade neuroendocrine tumors (35 patients). Median progression-free survival (PFS) was 31 months. The combined treatment has been well tolerated and has substantial tumor control rates [29].

PARP inhibitors may improve sensitization to radiation and alkylating treatments. Few data are available in mice for meningioma [30]. In addition, the tumor suppressor gene BAP1 is another biomarker for aggressive meningioma and may also represent a potential therapeutic target because BAP1 inactivation may increase tumor sensitivity to PARP inhibition. Recently, the antitumor effects of 177Lu-DOTA-TATE were enhanced by PARP inhibitors (talazoparib) in somatostatin receptor 2-expressing tumor models. These preclinical results suggest interest in the clinical evaluation of PARP inhibitors in combination with PRRT in SSTR2-expressing NETs. Human meningioma was not included in the panel of cell lines tested [31].

Immunotherapy is also becoming a potential alternative for HGM patients because of the immune microenvironment in meningiomas including microglia, macrophages, B-cells, and T-cells [32]. Meningiomas are not limited by the blood-brain barrier (BBB), making these tumors accessible to the peripheral immune cells. Primary research is focused on the use of monoclonal antibodies targeting PD1/PD-L1 to increase the anti-tumor immune response. A trial is underway to evaluate the therapeutic response after pembrolizumab and PRRT in grade 3 mNETs (metastatic-NETs).

Promising results in aggressive meningiomas can be expected using a combination of treatments. However, it is necessary to carefully define the population that might receive combination therapy instead of PRRT alone through a pertinent characterization of the tumor. Adverse hematologic events and peritumoral edema may be significant barriers to this type of therapy. Tumors with aggressive kinetics and high tumor burden are more likely to benefit from combination treatment because the expected effectiveness of treatment alone is low in this subgroup. 

Tolerance is one of the major issues with combination treatment. First, results from the controlNet trial—a non-comparative randomized open label phase II trial of PRRT +/− CAPTEM (capecitabin temozolomide) to determine the relative activity of adding CAPTEM to LuTate PRRT in patients with metastatic NETs (mNETs)—showed grade 3 adverse events in 22/32 CAPTEM patients (69%) vs. 5/13 (38%, PRRT); and 4/32 patients experienced one grade 4 event (13%) vs. 1/13 (8%), respectively [33].

One of the new tactics to reduce toxicity with combination treatment could be spacing out PRRT treatment. Parghane et al. in a study evaluating long-term outcome of a “sandwich chemo-PRRT” protocol in mNETs with both SSTR- and FDG- avid aggressive disease showed satisfactory results concerning the tolerance of this type of approach [34].

For future studies, two main situations can be particularly evaluated, one assessing the efficacy of PRRT in refractory progressive meningiomas and the other assessing the adjuvant efficacy of PRRT in risky or incomplete surgery in multifocal meningiomas in addition to EBRT.

This study has several limitations. First, the number of patients from the present study (*n* = 8) and incomplete data for 1 patient could introduce bias. Second, evaluation of long-term efficacy and toxicity was limited. Patient follow-up after the first cycle of PRRT ranges from 9 to 28 months. 

Furthermore, regarding response assessment, PFS-6 is suitable for the most aggressive meningiomas but is less relevant in intermediate meningiomas such as recurrent WHO grade II meningiomas. In order to detect early signals of anti-tumor drug activity or non-activity, we should have relied on the 3D MRI volume growth rate [25].

Third, in the literature review, the assessment of response using different criteria such as the 2D RANO criteria, RECIST, Mac Donald for meningioma, limited the relevance of comparisons between different studies [19]. Overall survival (OS) is only of interest in randomized studies, as there is a lack of comparative historical data. Other more clinical criteria, such as quality of life, seizure frequency, time to second-line treatment, or cognitive function to assess tumor response may be of interest.

One of the major limitations of our review is the inaccessibility of separate data for WHO grade II and III meningiomas from the Bartolomei et al. cohort (14 patients) and for all grades from Marincek et al. (14 patients). Another limitation of this study is the failure to include the number of cycles per patient in the analysis. We assume that the effect of cumulative doses has a positive impact on disease control, so it may influence outcomes. 

In addition, the adoption of the fifth edition of the WHO classification of CNS tumors in clinical practice, with the improved knowledge on the involvement of molecular alterations leads to tumor reclassifications. This has an impact in our study on the PFS according to the WHO grade. We reclassified a grade I meningioma (classified according to the 2007 WHO classification) to grade II meningioma. Studies should now reclassify meningiomas diagnosed before 2021 according to the latest classification and request molecular biomarkers to allow better comparison of data, to avoid potentially confounding effects on response and outcome when histologically similar but molecularly distinct tumors were studied together in the past.

## 5. Conclusions

PRRT may be a promising treatment for patients with refractory meningiomas, particularly those with grade I and II meningiomas. The treatment is well tolerated and stabilizes or slows tumor progression for at least a few months.

## Figures and Tables

**Table 1:** Patient characteristics.

	Age	Sex	Time from Diagnosis	Localization	WHO Grade	Treatment	Ki 67 Index Primitive	Ki 67 Index Recurrence	Neurological Deficit	ECOG PS
1	74	F	3 years	Multiple meningioma, metastasis	II	Surgery, EBRT, SRS, Sandostatine-Everolimus	15–20%	N/A	motor deficit, confusion	2
2	85	F	17 years	multiple meningioma; subcutaneous frontal lesion	II	Surgeryx3, SRSx2, EBRT	15%	N/A	visual disturbance	2
3	67	M	9 years	multiple meningioma	II	Surgeryx2, EBRT, SRS, Sandostatine-Everolimus	N/A	2%	left hemiparesis	2
4	72	M	19 years	multiple meningioma	II	Surgeryx2, EBRT, SRS, Sandostatine-Everolimus	N/A	25%	visual disturbance; frontal syndrome	1
5	60	M	9 years	multiple meningioma, metastasis	III; II	Surgeryx2, parotidectomy, EBRT	30–40%	10%	asthenia, attention deficit disorder	1
6	76	M	15 years	multiple meningioma	II	Surgeryx5, EBRT, SRSx3	10–15%	25; 20%	none	0
7	73	F	15 years	multiple meningioma	II	Surgeryx2, EBRT, SRS	10%	N/A	visual disturbance, ptosis, trigeminal neuralgia	1
8	67	M	11 years	multiple meningioma	II	Surgery, EBRT	N/A	N/A	left upper limb deficiency,	1

N/A: not available.3.2. Response Assessment MRI.

**Table 2:** Progression-Free Survival from initiation of PRRT.

	Krenning Score	WHO Grade	SUVmax Pretreatment	Cumulative Dose (GBq)	Best Radiologic Response	PFS 2D RANO Criteria
1	3	II	7.4	10.6	PD	2 months
2	3	II	26.3	29.6	SD	10 months
3	3	II	45	29.6	SD	17 months
4	2	II	14.5	29.6	SD	16 months
5	2	III; II	12.3	29.6	SD	not reached at 12 months
6	3	II	16	29.6	SD	not reached at 9 months
7	3	II	16.8	29.6	SD	N/A *
8	3	II	15.4	29.6	SD	not reached at 16 months

DOTATOC PET scans were graded using Krenning score (0 = no uptake; 1 = very low; 2 = equal to liver; 3 = greater than liver; 4 = greater than spleen. * MRI has not been performed during follow-up for patient 7. The 68Ga-DOTATOC lesion volumetry during follow-up was stable, but the patient was excluded from the PFS-6 analysis based on bidimensional RANO criteria on the MRI.

**Table 3:** 6-month Progression-Free Survival according to WHO-grade.

	Current Case Series	Müther et al. 2020 [17]	Parghane et al., 2019 [15]	Seystahl et al., 2016 [16]	Gerster-Gilliéron et al., 2016 [18]	Marincek et al., 2015 [14]	Bartolomei et al., 2009 [13]	Van Essen et al., 2006 [12]	Kreissl et al., 2012 [20] Hartrampf et al. 2020 [21]	Total Analysis
Radionuclide	^**177**^ **Lu-DOTATATE**	^**177**^ **Lu-DOTATATE**	^**177**^ **Lu-DOTATATE**	^**177**^ **Lu-DOTATATE or** ^**90**^ **Y-DOTATATE**	^**90**^ **Y-DOTATOC**	^**90**^ **Y-DOTATOC or** ^**177**^ **Lu DOTATOC**	^**90**^ **Y DOTATOC**	^**177**^ **Lu-DOTATATE**	**EBRT + 1 cycle** ^**177**^ **Lu-DOTA(TATE)/(TOC)**	
Patients	8 (7 patients included in the analysis)	7	5	20 (16 pts ^177^Lu-DOTATATE + 3 pts ^90^Y-DOTATATE + 1 combined); 19 patients included in the analysis	15	34 (66 cycles ^90^YDOTATOC et 8 cycles ^177^Lu DOTATOC)	29 treated; 28 patients included in the analysis	5	10	
Grade WHO-I pts	0	2 (28.5%)	N/A	4 analyzed (21%)	9 (60%)	5 (15%)	14 (48%)	N/A	6 (60%)	29 *
GI PFS6 %	0	100		100	100	N/A	78.6		100	**89.7**
Grade WHO-II pts	7 (100%)	5 (71.4%)	N/A	7 (35%)	2 (13%)	6 (18%)	9 (31%)	N/A	2 (20%)	21 **
GII PFS6 %	85.7	20		57	50	N/A	14.3 (GII + III 14 patients)		100	**57.1**
Grade WHO-III pts	0	0	N/A	8 (40%)	1 (6%)	3 (9%)	5 (21%)	3 (60%)	0	12 **
GIII PFS6 (%)				0	0	N/A	14.3 (GII + III 14 patients)	0		**0**
Unknown grade pts	0	0	5 (100%)	0	3 (20%)	20 (59%)	0	2 (40%)	2	10 **
Unknown grade PFS6 (%)			100		100	N/A		100	100	**100**
All grade PFS6 (%)	**85.7**	**42.8**	**100**	**42**	**86.7**	**N/A**	**46.4**	**40**	**100**	58.1 * (86 pts)
Radiological criteria for progression	*RANO*	*RANO*	RECIST 1.1	Macdonald	RECIST 1.1	N/A	SWOG	N/A	N/A	

PFS-6: progression-free survival at 6 months; EBRT: external beam radiotherapy; N/A: not available; Pts: patients; RECIST 1.1: Response Evaluation Criteria in Solid Tumors version 1.1; SWOG: Southwest Oncology Group; RANO: Response Assessment Neuro-Oncology; * studies from Marincek et al. [14] and Kreissl et al. [20] are not included in the analysis; ** studies from Marincek et al. [14], Bartolomei et al. [13], and Kreissl et al. [20] are not included in the analysis.

## Data Availability

Data can be retrieved from the corresponding author.
